# A Mendelian randomization study exploring the genetic associations between biliary system disorders and brain structural changes

**DOI:** 10.1097/MD.0000000000047616

**Published:** 2026-02-13

**Authors:** Run Qu, Qingfen Ruan, Ruiqin Han, Canmei Li, Yanhong Zhao, Yi Liang, Yuzhe Zhang

**Affiliations:** a School of Basic Medical Sciences, Dali University, Yunnan, China; b Department of Gastroenterology, The First Affiliated Hospital, Dali University, Dali, China; c Institute of Basic Medical Sciences, Chinese Academy of Medical Sciences & Peking Union Medical College, Beijing, China; d Department of Oncology, Dali Bai Autonomous Prefecture People’s Hospital, Yunnan, China; e Princess Margaret Cancer Centre, University Health Network, TMDT-MaRS Centre, Toronto, ON, Canada; f Yunnan Provincial Key Laboratory of Insect Medicine Research and Development, Yunnan, China.

**Keywords:** biliary system disorders, biliary–brain axis, cerebral cortex, Mendelian randomization (MR), subcortical volumes

## Abstract

The objective was to evaluate potential genetic associations between biliary system disorders and cortical and subcortical brain structural changes using Mendelian randomization analyses. This study focused on 4 diseases, including primary sclerosing cholangitis, cholecystitis, intrahepatic cholangiocarcinoma, and gallstone disease, and total bilirubin levels as indicators of biliary system disorders. Genome-wide association studies summary statistics from the ENIGMA consortium were utilized to assess the directional associations between these biliary system diseases and changes in brain structure, including the cerebral cortex (N = 51,665) and subcortical brain structures (N = 30,717). Inverse variance weighted was the primary method for conducting MR analysis. Furthermore, sensitivity analysis was conducted to evaluate the robustness of our findings. At the regional level, a decrease in the thickness of the pars opercularis was supposedly linked to genetically predicted total bilirubin levels (*P* = .00014). Decreased cortical thickness and surface area of the paracentral lobule were nominally associated with genetically predicted cholecystitis (*P*_TH_ = .024; *P*_SA_ = .042). Genetically predicted gallstone disease was correlated with an increased surface area of the transverse temporal gyrus (*P* = .023), while it was nominally associated with decreased thickness of the transverse temporal gyrus (*P* = .015), inferior parietal gyrus (*P* = .042), and middle temporal gyrus (*P* = .017). Furthermore, genetically predicted intrahepatic cholangiocarcinoma was associated with decreased thickness of the pars opercularis (*P* = .026) and surface area of the superior parietal gyrus (*P* = .013), while the thickness of the para hippocampal gyrus was increased without global weighted (*P* = .012) and with global weighted (*P* = .022). By comparison, genetically predicted primary sclerosing cholangitis was associated with a modest increase in the surface area of the para hippocampal gyrus, both with global weighted (*P* = .015) and without global weighted (*P* = .012), and was also linked to the increased thickness of the paracentral lobule (*P* = .016). No significant evidence of multiple testing effects or heterogeneity was observed. The research suggests an association between changes in the cerebral cortex and biliary system disorders, indicating an indirect impact of these abnormalities on cortical structures.

## 1. Introduction

The biliary system serves as a crucial conduit linking the liver and intestines, rendering it susceptible to severe inflammation and malignant conditions. Comprising the intrahepatic and extrahepatic bile ducts as well as the gallbladder, this system is responsible for facilitating bile drainage into the intestines. Given that hepatic accumulation of bile is toxic, biliary disorders can result in liver fibrosis and failure, necessitating liver transplantation in severe cases.^[1]^ Although biliary tract diseases are relatively rare, they are associated with high morbidity and mortality rates and lack effective cures, such as cholangiocarcinoma and primary sclerosing cholangitis (PSC).^[2]^ Furthermore, biliary tract diseases are closely associated with a variety of systemic diseases, including inflammatory bowel disease, metabolic syndrome, malignant tumors, and psychiatric disorders.^[3–5]^

An increasing number of studies have demonstrated that biliary tract diseases can result in a range of intestinal pathophysiological changes, such as compromised intestinal barrier function and heightened permeability, leading to endotoxemia or bacteremia, thereby exacerbating other complications.^[6]^ Up to 80% of PSC patients also present with inflammatory bowel disease, indicating a close association between its pathogenesis and damage to the intestinal barrier.^[7]^ Research has demonstrated a significant reduction in intestinal flora among cholecystitis patients compared with healthy controls.^[8]^ Furthermore, chronic intestinal inflammation resulting from biliary tract diseases can lead to impairment of both the enteric and central nervous systems while impacting blood-brain barrier function. This process increases susceptibility to neuroinflammation.^[9,10]^ Imbalances in gut flora regulation, along with central nervous system inflammation, are linked to various neurodegenerative conditions, including Alzheimer disease, Parkinson disease, multiple sclerosis, as well as neuropsychiatric disorders such as depression and anxiety disorders.^[11]^

The human cerebral cortex, as the outer gray matter layer of the brain, plays a crucial role in cognitive function. Brain morphology is one of the determinants of brain function, the basis for biomarker discovery, and a target for physical therapy.^[12]^ Therefore, understanding the association between biliary tract diseases and structural changes in the brain holds significant importance in uncovering disease mechanisms and exploring new therapeutic approaches.

Mendelian randomization (MR) uses genetic variants as instrumental variables (IVs) to evaluate the causal relationship between exposure and disease outcomes.^[13]^ Based on Mendel genetic law, the genetic effects of gene phenotypes are not influenced by traditional confounding factors, and genetic variations remain unchanged after individual birth, thus avoiding bias in reverse causation. Therefore, MR can effectively avoid confounding bias and reverse causality issues in traditional observational studies.^[14]^ In recent years, large-scale genome-wide association studies (GWAS) have rapidly identified common genetic variations,^[15]^ providing new avenues to explore genetic links between biliary disorders and cortical and subcortical structures. We used summary statistics data from large GWAS to address this issue, using single nucleotide polymorphisms (SNPs) associated with biliary tract disease phenotypes as IVs.

In this study, we used an MR analysis to investigate the potential associations between biliary diseases [including total bilirubin (TBIL), PBC, cholecystitis, intrahepatic cholangiocarcinoma (ICC), and gallstone disease (GSD)] and variations in brain structure [surface area (SA)/thickness (TH) and subcortical volumes]. The findings provide new insights into the link between biliary tract system diseases and structural alterations in the cerebral cortex.

## 2. Materials and methods

### 2.1. Data sources

#### 2.1.1. Exposure data

The data on biliary tract diseases were obtained from the UK Biobank (http://www.nealelab.is/uk-biobank) and FinnGen (r10.finngen.fi) databases. Summary statistics for PSC in individuals of European descent were extracted from the FinnGen database, including 1843 cases and 361,641 controls (ICD code ICD:10 K83.0), as well as summary statistics for cholecystitis, with 4697 cases and 361,641 controls (ICD code ICD:10 K81), and ICC, with 2764 cases and 10,475 controls (ICD code ICD:10 C22.1). Additionally, summary statistics for GSD were extracted from the UK Biobank database, including 393,104 cases and 1268,638 controls, as well as TBIL levels in serum with 468,748 cases and 485,197 controls. Changes in TBIL are considered a predictive indicator for acute cholecystitis and symptomatic gallstone disease. Previous studies have indicated that elevated plasma bilirubin levels are associated with an increased risk of symptomatic biliary tract disease.^[16]^

#### 2.1.2. Outcome data

The data on subcortical brain regions and the cerebral cortex (TH and SA) were obtained from the ENIGMA consortium (https://enigma.ini.usc.edu/). Data from the cerebral cortex, primarily of European origin, were collected using magnetic resonance imaging (MRI) from 51,665 individuals across 60 cohorts worldwide. This study utilized findings from a meta-analysis involving individuals of European ancestry.^[17]^ The Desikan–Killiany atlas was employed to delineate the 34 regions of interest, with specific structures identified within deep cortical trenches to establish regional boundaries.^[18]^

Data on subcortical brain regions were obtained from the MRI data of 30,717 individuals across 50 cohorts. This dataset included the volumes of 7 subcortical regions and the intracranial volume.^[19]^ With or without global weighted estimation of the entire brain, MR analyses were conducted for TBIL, PSC, cholecystitis, ICC, and GSD, encompassing the TH and SA of the entire cerebral cortex, 34 functionally specialized cortical regions, as well as subcortical brain regions. The results amounted to 369.

### 2.2. IV selection

To investigate the association between biliary tract diseases and the cortical and subcortical brain structures, we selected 5 sets of genetic IVs: index SNPs for PSC, index SNPs for cholecystitis, index SNPs for ICC, index SNPs for GSD, and index SNPs for TBIL. The criteria for selecting genetic variables are as follows: GWAS-related *P*-value of 5 × 10^−6^; linkage disequilibrium (*r*^2^ < 0.001, region width of 10,000 kb), ensuring the independence and correlation of SNPs^[20]^; the F statistic may be computed using the following formula: *F* = *R*^2^(N *−* 2)/(1 *−* *R*^2^) (where *R*^2^ represents the IV exposure variance and N is the sample size). *F* > 10 is sufficient to counteract bias from weak IVs.^[21]^ Before each MR analysis, *P* > 5 × 10^−6^ of SNPs associated with brain cortical structure were excluded, and the MR pleiotropy residual sum and outlier (MR-PRESSO) was applied to remove potential outliers. This test was most suitable when the level of pleiotropy in IVs is *P* > .05.^[22]^

To ensure robust MR analysis, the selection of IVs must adhere to 3 fundamental hypotheses: the IVs must be significantly related to the exposure factor (association hypothesis); the IVs must remain unaffected by any additional variables that could influence the outcome (independence hypothesis); and the IVs cannot influence the outcome in any other way, and their influence is solely through the exposure factor, which determines the likelihood of the outcome occurring (exclusivity hypothesis).

### 2.3. MR analysis

In this study, inverse variance weighted (IVW), MR-Egger regression, and weighted median methods in the TwoSample MR package were used. The MR analysis was performed for associations between TBIL, PSC, cholecystitis, ICC, and GSD and changes in brain structure (cortex and subcortical regions). The primary analysis method is the IVW method, which provides the most accurate estimates by taking the weighted average of the causal effect sizes of all the IVs. However, IVW ignores invalid IVs and pleiotropic effects. Therefore, using MR-Egger and weighted median as supplementary methods to the IVW estimator can provide more reliable estimates in a wider range of studies.

IVW is considered the standard method for summarizing data in MR. MR-Egger is used to detect and correct bias in IVs and assess horizontal pleiotropy, with fitted weights determined by the reciprocal of the outcome variance.^[23]^ The weighted median method offers the advantages of consistent and robust assessments in MR analysis, especially when more than 50% of the weights come from valid IVs.^[24]^

### 2.4. Sensitivity analysis and pleiotropy analysis

In the sensitivity analysis, Cochran *Q* statistic (from the TwoSample MR package) was calculated using both IVW and MR-Egger regressions. Heterogeneity was assessed with Cochran *Q* test, where *P* > .05 indicates no significant heterogeneity and *P* ≤ .05 indicates significant heterogeneity. In the pleiotropy analysis, the intercept terms from both the MR-Egger regression and MR-PRESSO test were used to assess horizontal pleiotropy of the SNPs. A near-zero intercept in the MR-Egger regression suggests the absence of horizontal pleiotropy.^[25]^ Further assessment of horizontal pleiotropy was conducted using the MR-PRESSO test, where an outlier SNP was identified and subsequently excluded from further analysis.

**Figure 1.**
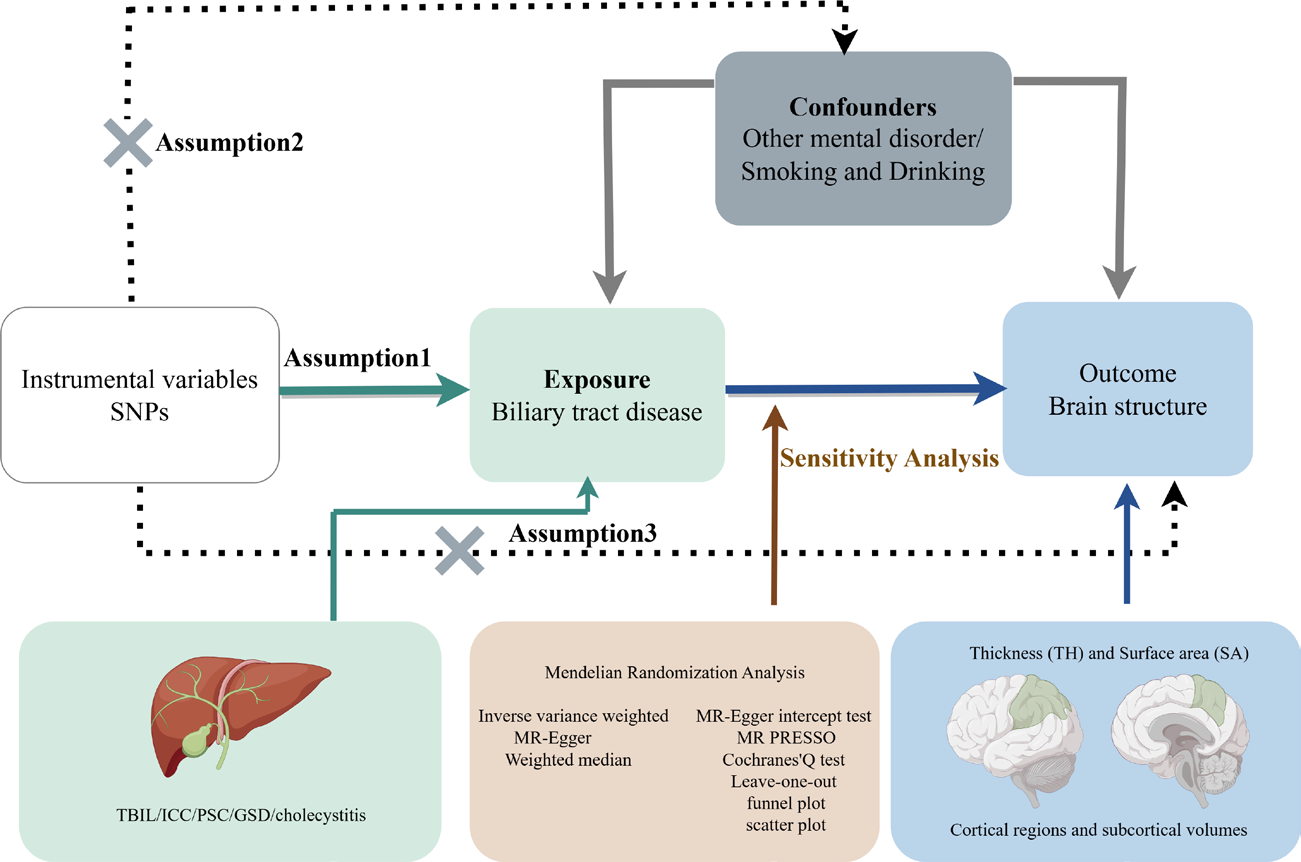
The study is designed to investigate the association between biliary diseases and cortical and subcortical brain regions (cortical surface area, thickness, and subcortical volumes) based on the principles of Mendelian randomization. GSD = gallstone disease, ICC = intrahepatic cholangiocarcinoma, PSC = primary sclerosing cholangitis, SNP = single nucleotide polymorphism, TBIL = total bilirubin.

Additionally, to control type I error inflation due to multiple comparisons, we applied the Bonferroni correction separately for the whole cortex and global-level analyses. Across the whole cortex, associations with *P* < 1.35 × 10^−4^ were considered statistically significant after Bonferroni correction, whereas those with 0.05 > *P* ≥ 1.35 × 10^−4^ were classified as nominally significant and interpreted as providing suggestive, but not definitive, evidence of potential causal relationships. For global-level analyses, the Bonferroni-corrected 2-sided significance threshold was set at *P* < .01 (α = 0.05/5, corresponding to 5 outcomes). All statistical analyses were conducted using the Mendelian Randomization package (version 0.9.0) and the TwoSampleMR package (version 0.5.8) in R (version 4.3.2). The research framework diagram is shown in Figure 1.

### 2.5. Ethical approval

This study was based exclusively on publicly available GWAS summary statistics from previously published studies. All contributing studies had received ethical approval from their respective institutional review boards and obtained informed consent from participants. As no individual-level data were used and no new human participants were recruited, additional ethical approval and informed consent were not required for this study.

## 3. Results

### 3.1. Selection and validation of IVs

For genetically predicted TBIL, 233 SNPs were initially assessed; 14 SNPs were retained as instruments for PSC, 7 for cholecystitis, 77 for ICC, and 38 for GSD. Detailed information on all IVs, including individual *F*-values, harmonization details, and excluded SNPs, is provided in Tables S3 and S4, Supplemental Digital Content, https://links.lww.com/MD/R354. The *F*-statistics for all IVs exceeded 10, indicating a minimal risk of weak instrument bias. Potential pleiotropic SNPs were identified using the PhenoScanner tool (http://www.phenoscanner.medschl.cam.ac.uk/) and excluded if associated with established risk factors (obesity, smoking, alcohol consumption, or neuropsychiatric disorders), including rs8192589, rs2840001, rs1082218, rs6831352, rs9845788, and rs6910668.

**Figure 2.**
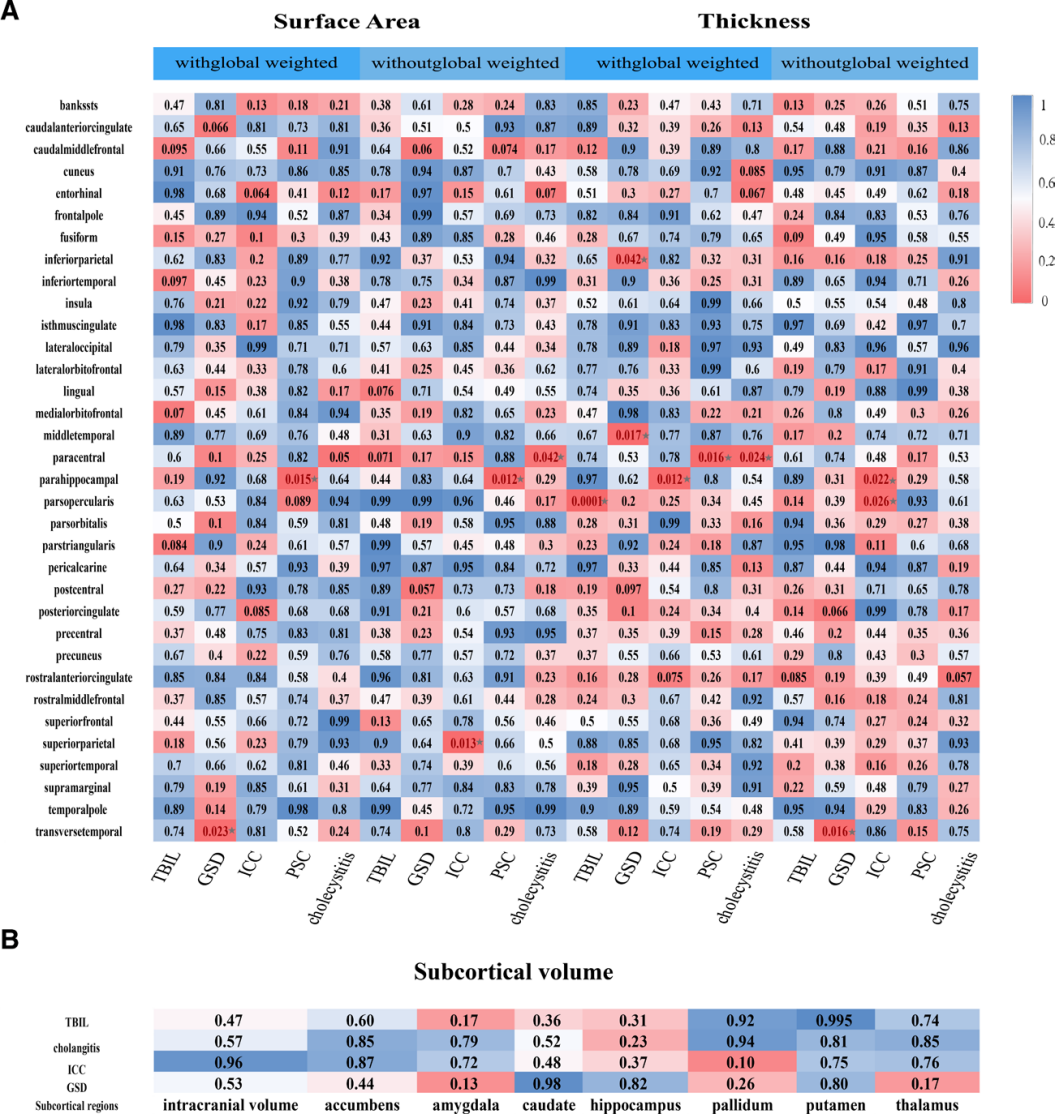
Heatmaps from inverse variance weighted (IVW) analyses assessing the associations between TBIL, GSD, ICC, PSC, and cholecystitis with cortical thickness (TH) and surface area (SA), as well as subcortical volumes. (A) *P*-values for cortical surface area (SA, left) and cortical thickness (TH, right) across 34 cortical regions, shown with and without global adjustment. (B) *P*-values for subcortical volumes. Each square displays the *P*-value for a specific biliary trait–brain structure; warmer colors (red) indicate smaller *P*-values and cooler colors (blue) indicate larger *P*-values. A *P-value < .05 represents nominal evidence of association. GSD = gallstone disease, ICC = intrahepatic cholangiocarcinoma, PSC = primary sclerosing cholangitis, TBIL = total bilirubin.

**Figure 3.**
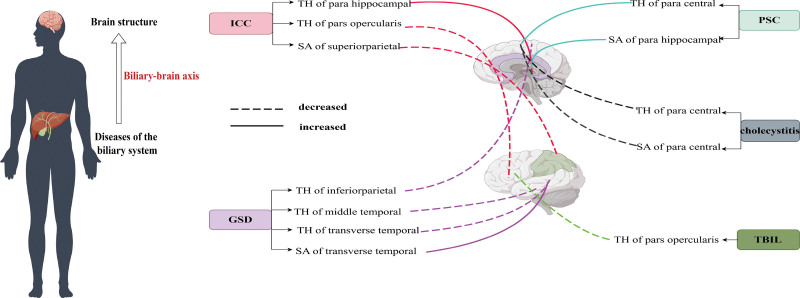
This figure illustrates the conceptual framework of the biliary–brain axis and summarizes the nominal associations identified between biliary system diseases and cortical structural changes. Solid lines indicate nominal associations with increased cortical thickness (TH) or surface area (SA), whereas dashed lines indicate nominal associations with decreased TH or SA. GSD = gallstone disease, ICC = intrahepatic cholangiocarcinoma, PSC = primary sclerosing cholangitis, TBIL = total bilirubin.

The MR study evaluated the suggestive associations between genetically predicted TBIL, PSC, cholecystitis, ICC, and GSD with subcortical brain structures, as well as with cortical SA and TH, both with and without global weighting. An overview of the IVW effect estimates for all biliary trait–brain structure pairs is presented in Figure 2, where nominal associations (*P < .05) are highlighted by the color scale. Figure 3 illustrates the framework of the biliary–brain axis and summarizes the nominal associations identified between biliary system diseases and cortical structural changes.

### 3.2. Global cortical and subcortical structures

At the global level, no causal relationship was shown between biliary tract disease and cortical SA and TH (Table S1, Supplemental Digital Content, https://links.lww.com/MD/R355). However, in the PSC analysis of genetic prediction, we detected a heterogeneous outlier (rs137977673), which we subsequently excluded. Ultimately, we selected 4 sets of genetic instruments. In addition, biliary tract diseases (including TBIL, ICC, GSD, and cholecystitis) did not have significant associations with subcortical regions (Table S2, Supplemental Digital Content, https://links.lww.com/MD/R355). Sensitivity analysis showed no horizontal pleiotropy or heterogeneity in the global cerebral cortex (TH and SA) and subcortical areas.

### 3.3. Association of PSC with cortical structures

In regional analyses, genetically predicted PSC was nominally associated with an increased surface area of the para hippocampal gyrus [β = 3.3192 mm^2^, 95% confidence interval (CI): 0.6455–5.9928 mm^2^, *P* = .015], an increased thickness of the paracentral lobule (β = 0.0050 mm, 95% CI: 0.0009–0.0090 mm, *P* = .016), and an increased surface area of the para hippocampal gyrus without global weighted (β = 4.0990 mm^2^, 95% CI: 0.8830–7.3150 mm^2^, *P* = .012). Additionally, a possible association between PSC and the thickness of the paracentral lobule (β = 0.0059 mm, 95% CI: 0.0008–0.0109 mm, *P* = .023), as well as the surface area of para hippocampal gyrus without global weighting (β = 4.9589 mm^2^, 95% CI: 0.6552–9.2626 mm^2^, *P* = .024), was supported by the weighted median results.

### 3.4. Association of cholecystitis with cortical structures

Genetically predicted cholecystitis was nominally associated with a decrease in the SA and TH of the paracentral lobule (β_TH_ = −0.0066 mm, 95% CI_TH_: −0.0123 to −0.0008 mm, *P*_TH_ = .024; β_SA_ = −8.1917 mm^2^, 95% CI_SA_: −16.0723 to −0.3111 mm^2^, *P*_SA_ = .042). Furthermore, the weighted median analysis corroborated this association (β_TH_ = −0.0079mm, 95% CI_TH_: −0.0153 to −0.0006 mm, *P*_TH_ = .035; β_SA_ = −11.8286 mm^2^, 95% CI_SA_: −22.3232 to −1.3341 mm^2^, *P*_SA_ = .027).

### 3.5. Association of ICC with cortical structures

Genetically predicted ICC was nominally associated with a decreased surface area of the superior parietal gyrus (β = −9241.88 mm^2^, 95% CI: −16,563.2 to −1920.52 mm^2^, *P* = .013). Furthermore, ICC was found to be nominally associated with an increased thickness of the para hippocampal gyrus (β = 3.0153 mm, 95% CI: 0.4348–5.5958 mm, *P* = .022), decreased thickness of the pars opercularis (β = −1.4205, 95% CI: −2.6720 to −0.1690 mm, *P* = .026), and increased thickness of the para hippocampal gyrus without global weighted (β = 3.0153 mm, 95% CI: 0.7174–5.7805mm, *P* = .012). Concurrently, the weighted median results confirmed the associations of an ICC with the surface area of the superior parietal gyrus and thicknesses of the pars opercularis and para hippocampal gyrus. The thickness of the para hippocampal gyrus with global weighted (β = 4.2234 mm, 95% CI: 0.5973–7.8496 mm, *P* = .022) and without global weighted (β = 5.7631mm, 95% CI: 2.1187–9.4077 mm, *P* = .0019), the thickness of pars opercularis (β = −2.2070, 95% CI: −3.9522 to −0.4619, *P* = .013), the surface area of the superior parietal gyrus (β = −11,147.6 mm^2^, 95% CI: −20,384.8 to −1910.41 mm^2^, *P* = .018).

### 3.6. Association of GSD with cortical structures

Genetically predicted GSD was nominally associated with the SA and TH of the transverse temporal gyrus (β_TH_ = −0.3087 mm, 95% CI_TH_: −0.5596 to −0.0578 mm, *P*_TH_ = .015; β_SA_ = 69.3405 mm^2^, 95% CI_SA_: 9.5868–129.0942 mm^2^, *P*_SA_ = .023), Concurrently, it was related to decreased thickness of the inferior parietal gyrus (β = −0.0911 mm, 95% CI: −0.1788 to −0.0034 mm, *P* = .042) and middle temporal gyrus (β = −0.1516 mm, 95% CI: −0.2765 to −0.0267 mm, *P* = .017). Despite significant deviations in the IVW results, the weighted median approach, and the MR-Egger method, consistency in the β direction was maintained.

### 3.7. Association of TBIL with cortical structures

Genetically predicted TBIL was nominally associated with a decreased thickness of the pars opercularis (β = −0.0087 mm, 95% CI: −0.0140 to −0.0034 mm, *P* = .0014), and weighted median analysis results also supported the association (β = −0.0114 mm, 95% CI: −0.0201 to −0.0027 mm, *P* = .011).

**Figure 4.**
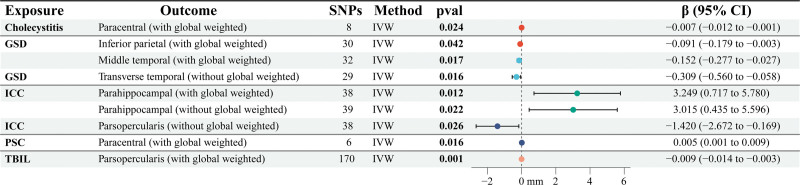
Forest plots of inverse variance weighted (IVW) effect estimates for the associations between genetically predicted TBIL, PSC, cholecystitis, ICC, and GSD and regional cortical thickness (TH). Horizontal lines represent the 95% confidence intervals, and markers indicate the β estimates (mm). Estimates to the right of the zero line indicate increased cortical thickness, while those to the left indicate decreased cortical thickness. GSD = gallstone disease, ICC = intrahepatic cholangiocarcinoma, PSC = primary sclerosing cholangitis, SNP = single nucleotide polymorphism, TBIL = total bilirubin.

**Figure 5.**
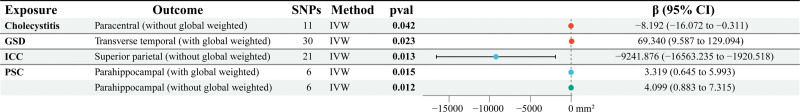
Forest plots of inverse variance weighted (IVW) effect estimates for the associations between genetically predicted TBIL, PSC, cholecystitis, ICC, and GSD and regional cortical surface area (SA). Horizontal lines represent the 95% confidence intervals, and markers indicate the β estimates (mm^2^). GSD = gallstone disease, ICC = intrahepatic cholangiocarcinoma, PSC = primary sclerosing cholangitis, SNP = single nucleotide polymorphism, TBIL = total bilirubin.

A detailed graphical summary of the IVW estimates for the TH and SA for all 5 biliary traits (TBIL, ICC, PSC, GSD, and cholecystitis) is provided in Figures 4 and 5. Figure 4 presents forest plots for regional cortical TH, whereas Figure 5 presents the corresponding plots for regional cortical SA. After applying the Bonferroni correction, all of these findings remained nominally significant.

### 3.8. Sensitivity analyses

In sensitivity analyses (Table 1), Cochrane *Q* test, based on both MR-Egger and IVW, showed that significant heterogeneity was observed in TBIL, PSC, ICC, GSD, and cholecystitis (*P* > .05). Furthermore, there was no indication of a significant intercept, indicating the lack of level pleiotropy between biliary tract illness and the cerebral cortex. Furthermore, leave-one-out analyses demonstrated that no single SNP disproportionately influenced the overall estimates (Fig. S2, Supplemental Digital Content, https://links.lww.com/MD/R355), and no outliers were detected in funnel plot or scatter plot inspections (Figs. S1 and S3, Supplemental Digital Content, https://links.lww.com/MD/R355). We also performed additional robustness checks, including MR-PRESSO tests, which further confirmed the stability and reliability of the results (Table S5, Supplemental Digital Content, https://links.lww.com/MD/R354).

**Table 1:** Significant IVW estimates were observed for genetically predicted TBIL, ICC, PSC, GSD, and cholecystitis concerning cerebral cortical structure (TH and SA).

Exposures	Outcomes	Cochrane test		MR-Egger intercept	
		*Q*-value	*P*_Q_	Intercept	*P*_Intercept_
TBIL	TH of pars opercularis with global weighted	145.6430	.90	0.000037	.71
PSC	SA of para hippocampal gyrus with global weighted	3.0234	.70	0.5820	.44
	TH of paracentral lobule with global weighted	5.3669	.37	−0.00013	.26
	SA of para hippocampal gyrus without global weighted	2.7980	.73	0.4490	.61
ICC	TH of para hippocampal gyrus with global weighted	37.8082	.43	0.00066	.71
	TH of para hippocampal gyrus without global weighted	33.5946	.67	0.00024	.89
	TH of pars opercularis without global weighted	22.8389	.97	0.00005	.95
	SA of superior parietal gyrus without global weighted	10.1661	.96	0.2733	.96
GSD	TH of inferior parietal gyrus with global weighted	24.2418	.72	0.00011	.59
	TH of middle temporal gyrus with global weighted	22.2051	.88	0.000006	.84
	SA of transverse temporal gyrus with global weighted	18.1558	.94	0.0730	.62
	TH of transverse temporal gyrus without global weighted	23.7533	.78	−0.00009	.82
Cholecystitis	TH of paracentral lobule with global weighted	3.1965	.87	−0.00078	.53
	SA of paracentral lobule without global weighted	8.0934	.62	1.9202	.29

GSD = gallstone disease, ICC = intrahepatic cholangiocarcinoma, IVW = inverse variance weighted, PSC = primary sclerosing cholangitis, SA = surface area, SNP = single nucleotide polymorphism, TBIL = total bilirubin, TH = thickness.

## 4. Discussion

Previous studies have reported potential associations between biliary system disorders and cortical and subcortical brain regions. However, the underlying genetic relationships between these traits remain poorly understood. To address this gap, this study is the first to use an MR analysis to investigate putative associations between biliary tract disease-related exposure factors, and cortical and subcortical brain structures. Cortical and subcortical regions form interconnected circuits that coordinate motor, learning, and memory processes, and alterations in these structures have been implicated in abnormal behaviors and in the development of neurological disorders, including Alzheimer disease, Parkinson disease, and amyotrophic lateral sclerosis.

In this study, GSD liability was inversely associated with the TH of the middle temporal, inferior parietal, and transverse temporal regions, whereas the SA of transverse temporal lobes showed a positive association. Although the observed stronger association between the surface area of the transverse temporal lobe and GSD than anticipated may have been influenced by the limited number of SNPs in the IV, our findings indicate a connection between GSD and cortical thinning, particularly in the temporal and parietal lobes. GSD has been linked to systemic metabolic and inflammatory perturbations (such as IL-6 and TNF-α), which could influence brain structure through neuroimmune or vascular pathways.^[26,27]^ The pathways provide a biologically plausible mechanism for how GSD could influence brain structure. The temporal lobe plays a critical role in facial recognition and complex visual development. Reduced thickness of the temporal lobule is a prominent feature observed in individuals with dementia or cognitive impairment, with memory impairment frequently linked to temporal lobe dysfunction.^[28]^ Furthermore, the reduction in temporal lobe thickness is closely associated with cognitive decline, the onset of Alzheimer disease, and the development and progression of epilepsy. Similarly, the parietal lobe responsible for integrating, processing, and interpreting sensory data is crucial for controlling motor actions based on sensory input.^[29]^ Patients with parietal lobe damage may exhibit dyslexia, apraxia (the inability to exercise), and sensory disturbances. Evidence suggests that individuals with cognitive impairment show atrophy of the inferior parietal lobule, with the degree of atrophy positively correlated with the severity of cognitive impairment in dementia patients.^[30,31]^ These associations highlight a potential link between GSD-related metabolic and inflammatory processes and brain structural variation, while the need for further research remains.

Cholecystitis liability showed inverse associations with both TH and SA of the paracentral lobule. The paracentral lobule is a medial extension of the precentral and postcentral gyri and is implicated in sensorimotor control as well as behavioral regulation.^[32]^ A study has suggested an abnormal paracentral lobule structure or function and suicidal behavior in individuals with mood disorders.^[33]^ Another study has suggested that, based on voxel-based morphometry, reduced paracentral lobule volume may be associated with features exhibited by patients with autism spectrum disorders.^[34]^ Further research is needed to determine whether cholecystitis induces structural or functional changes in the paracentral lobule.

ICC liability demonstrated inverse correlation with both the TH of the pars opercularis and the SA of the superior parietal lobule. Central nervous system metastases from cholangiocarcinoma are extremely rare but can cause neurological symptoms when they occur, such as headaches, slowed mobility, and blurred consciousness in patients.^[35]^ In addition, brain imaging studies using Fluorodeoxyglucose combined with computed tomography have revealed mild cortical atrophy, ventricular dilatation, and slight focal thickening of the cerebellar pia mater in patients with ICC.^[36]^ Importantly, our MR findings should not be conflated with metastatic involvement; they more likely reflect shared genetic and biological pathways (e.g., immune regulation and inflammation) that could simultaneously influence cancer susceptibility and brain structural variation.

Elevated TBIL was inversely associated with the TH of the pars opercularis. Previous studies suggest that when bilirubin reaches a certain threshold, it may induce neurotoxicity and increase the risk of Alzheimer disease, providing directional evidence for the potential harm of high bilirubin levels.^[37]^ Additionally, bilirubin levels have been identified as a significant biomarker for depression risk among patients with ischemic stroke.^[38]^ Diffusion kurtosis imaging studies have observed increased kurtosis in regions such as the globus pallidus, thalamus, frontal lobe, temporal lobe, and hippocampus in neonatal bilirubin encephalopathy, which is correlated with serum bilirubin levels.^[39]^ These findings suggest that bilirubin-related brain injury exhibits regional selectivity. Given the direct ventral medial projections to the prefrontal cortex-amygdala pathway, the pars opercularis, located in the prefrontal cortex, plays a critical role in emotion regulation. Thinner pars opercularis may be present in patients with elevated bilirubin levels, which might potentially worsen anxiety and depression and interfere with mood regulation.^[40]^ The association between the two may be influenced by various potential confounding factors, including liver function and hemolysis. Therefore, further validation through larger prospective cohort studies and experiments is needed.

Furthermore, our study identified a positive correlation between PSC and the SA of the para hippocampal gyrus, as well as the TH of the paracentral lobule. Previous studies have reported that patients with PSC exhibit alterations in the structure or function of deep gray matter regions, which have been associated with symptoms such as fatigue, depression, and anxiety.^[41]^ However, the observed increases in SA and TH findings differ from prior expectations based on pathophysiological assumptions. These contradictory results could potentially reflect artifacts, false positives due to multiple comparisons, limitations in sample size, or other unaccounted confounding factors. Therefore, the observed positive associations should be interpreted with caution. In conclusion, our findings suggest that PSC may be associated with regional structural variations in the para hippocampal gyrus and paracentral lobule. Nevertheless, further clinical studies involving larger cohorts are necessary to replicate these observations, elucidate their clinical significance, and ascertain potential causal relationships.

In conclusion, our study indicates that cortical TH and SA in specific functional regions are associated with several key indicators of biliary tract disorders, including elevated TBIL, ICC, GSD, and cholecystitis. We observed negative associations between genetic liability to biliary tract disease and TH/SA in the pars opercularis, superior parietal lobule, paracentral lobule, inferior parietal lobule, middle temporal gyrus, and transverse temporal gyrus. In contrast, PSC was associated with increased TH of the paracentral lobule and increased SA of the para hippocampal gyrus.

In MRI research, integrating multimodal imaging sequences can facilitate a comprehensive assessment of brain structures. However, the data fusion process may introduce measurement variability, potentially affecting the accuracy of structural analyses and the reliability of result interpretation. In the present study, we observed that the MR-derived effect sizes for PSC, cholecystitis, TBIL, and cortical thickness in specific regions, particularly in the paracentral lobule and pars opercularis, consistently remained small (β ranging from −0.0087 to 0.0050 mm).

Previous studies have suggested that regional-specific variability can limit the accuracy of structural measurements in certain brain areas, with cortical thickness showing higher general variability, particularly in regions such as the inferior temporal cortex and the frontal pole.^[42]^ This means that in these regions, even small statistical differences might be compounded by measurement instability, making it difficult to fully rule out variability-driven effects. Additionally, factors such as scanner stability, image resolution, and differences in acquisition planes may contribute to measurement error, potentially amplifying or obscuring the true subtle effects.^[43]^ While the paracentral lobule and pars opercularis are associated with PSC, cholecystitis, and TBIL (*P* < .05), the small effect sizes observed are still highly susceptible to confounding by measurement noise and systematic errors, necessitating a cautious interpretation. Future studies incorporating independent replication, standardized imaging pipelines, and optimized scanner protocols are necessary to determine whether these subtle structural differences translate into measurable neuropsychiatric outcomes.

Our study has several advantages. First, in comparison with other observational studies, we employed the MR analysis to investigate the association between biliary tract disease and cortical and subcortical brain structures to mitigate and reduce confounding variables and prevent reverse causality. Second, the findings could have important implications for public health policy, especially in the early prevention and diagnosis of diseases of the biliary system and neuropsychiatric system. Finally, we used 5 sets of genetic tool variables to represent biliary system diseases, thereby improving the accuracy of the MR analysis results.

However, our analysis also has limitations. First, fully excluding the impacts of potential level pleiotropy and heterogeneity, which could bias estimates of causal effects, was challenging.^[25]^ Nonetheless, this was unlikely given that most of the enrolled population was of European ancestry, and no horizontal pleiotropy effect was observed in the MR-Egger intercept. Second, our study’s estimates of MR-Egger and weighted median were not statistically significant. Ideally, significant results from all 3 methods would be preferable. Nevertheless, we prioritize maintaining consistency in the direction of β. Therefore, despite the deviation of the 3 methods, the analysis results are still reliable. Third, compared with others, patients with biliary system diseases may have taken preventive measures, such as preventing central nervous system infections by maintaining the blood-brain barrier, which may have contributed to the biased results. However, genetic susceptibility studies are not influenced by interactions with other factors, which may mitigate bias. Fourth, a major limitation of the present study is the reliance on GWAS summary statistics derived exclusively from populations of European ancestry, reflecting constraints in currently available data. This population stratification raises concerns regarding the generalizability of our findings, particularly given established racial and ethnic differences in the epidemiology of PSC and cholangiocarcinoma. Incidence rates, risk factors, and disease progression patterns vary substantially across ancestral groups, with PSC being more prevalent in individuals of Northern European descent, while cholangiocarcinoma exhibits significantly higher incidence rates in East Asian populations. Consequently, genetic associations identified in European-ancestry cohorts may not fully capture or apply to the allelic architecture or effect sizes in diverse populations. Caution is therefore warranted when interpreting and extrapolating these results to non-European individuals.

## 5. Conclusion

In our comprehensive MR analysis, we identified potential associations between biliary tract diseases and specific brain structures, offering preliminary genetic evidence consistent with a potential gut–brain axis while not establishing definitive causal relationships. Furthermore, biliary system diseases that cause dysbiosis of the gut microbiota and chronic intestinal inflammation can disrupt gastrointestinal regulation. This study provides important clues to the genetic relationship between biliary tract disease and the cerebral cortex. In our subsequent work, we will further investigate the involvement of biliary system disorders in cerebral cortex pathogenesis through in vivo and in vitro experiments, exploring their specific role in disease progression.

## Acknowledgments

We express our gratitude to UK Biobank, FinnGen, and the ENIGMA Consortium for providing the GWAS summary statistics.

## Author contributions

**Conceptualization:** Run Qu, Qingfen Ruan, Yuzhe Zhang.

**Formal analysis:** Qingfen Ruan, Ruiqin Han.

**Investigation:** Run Qu, Yuzhe Zhang.

**Methodology:** Qingfen Ruan.

**Resources:** Run Qu, Yuzhe Zhang.

**Supervision:** Canmei Li, Yi Liang, Yuzhe Zhang.

**Writing – original draft:** Run Qu.

**Writing – review & editing:** Run Qu, Qingfen Ruan, Ruiqin Han, Canmei Li, Yanhong Zhao, Yi Liang, Yuzhe Zhang.

## Supplementary Material





## References

[1] Kurial SNT Willenbring H. Emerging cell therapy for biliary diseases. Science. 2021;371:786–7. 33602846 10.1126/science.abg3179 PMC8549489

[2] Prokopič M Beuers U. Management of primary sclerosing cholangitis and its complications: an algorithmic approach. Hepatol Int. 2021;15:6–20. 33377990 10.1007/s12072-020-10118-x PMC7886831

[3] Kim Y Oh CM Ha E Park SK Jung JY Ryoo J-H. Association between metabolic syndrome and incidence of cholelithiasis in the Korean population. J Gastroenterol Hepatol. 2021;36:3524–31. 34097775 10.1111/jgh.15568 PMC9291184

[4] Jukić T Margetić BA Jakšić N Boričević V. Personality differences in patients with and without gallstones. J Psychosom Res. 2023;169:111322. 37018955 10.1016/j.jpsychores.2023.111322

[5] Shen H He M Lin R. PLEK2 promotes gallbladder cancer invasion and metastasis through EGFR/CCL2 pathway. J Exp Clin Cancer Res. 2019;38:247. 31182136 10.1186/s13046-019-1250-8 PMC6558801

[6] Giacometti A Cirioni O Ghiselli R. Administration of protegrin peptide IB-367 to prevent endotoxin induced mortality in bile duct ligated rats. Gut. 2003;52:874–8. 12740345 10.1136/gut.52.6.874 PMC1773671

[7] Dyson JK Beuers U Jones DEJ. Primary sclerosing cholangitis. Lancet. 2018;391:2547–59. 29452711 10.1016/S0140-6736(18)30300-3

[8] Kummen M Holm K Anmarkrud JA. The gut microbial profile in patients with primary sclerosing cholangitis is distinct from patients with ulcerative colitis without biliary disease and healthy controls. Gut. 2017;66:611–9. 26887816 10.1136/gutjnl-2015-310500

[9] Bjarnason I Hayee B Pavlidis P. Contrasting pattern of chronic inflammatory bowel disease in primary and autoimmune sclerosing cholangitis. EBioMedicine. 2015;2:1523–7. 26629548 10.1016/j.ebiom.2015.08.041 PMC4634318

[10] Holland AM Bon-Frauches AC Keszthelyi D Melotte V Boesmans W. The enteric nervous system in gastrointestinal disease etiology. Cell Mol Life Sci. 2021;78:4713–33. 33770200 10.1007/s00018-021-03812-y PMC8195951

[11] Seo DO Holtzman DM. Gut microbiota: from the forgotten organ to a potential key player in the pathology of Alzheimer’s disease. J Gerontol A Biol Sci Med Sci. 2020;75:1232–41. 31738402 10.1093/gerona/glz262 PMC7302187

[12] Pang JC Aquino KM Oldehinkel M. Geometric constraints on human brain function. Nature. 2023;618:566–74. 37258669 10.1038/s41586-023-06098-1 PMC10266981

[13] de Ruiter SC Schmidt AF Grobbee DE. Sex-specific Mendelian randomisation to assess the causality of sex differences in the effects of risk factors and treatment: spotlight on hypertension. J Hum Hypertens. 2023;37:602–8. 37024639 10.1038/s41371-023-00821-1 PMC10403357

[14] Davey SG Hemani G. Mendelian randomization: genetic anchors for causal inference in epidemiological studies. Hum Mol Genet. 2014;23:R89–98. 25064373 10.1093/hmg/ddu328 PMC4170722

[15] Burgess S Butterworth A Thompson SG. Mendelian randomization analysis with multiple genetic variants using summarized data. Genet Epidemiol. 2013;37:658–65. 24114802 10.1002/gepi.21758 PMC4377079

[16] Katz MH. Bilirubin, gallstones, and mendelian randomization. JAMA Intern Med. 2013;173:1229. 23752680 10.1001/jamainternmed.2013.6568

[17] Grasby KL Jahanshad N Painter JN. The genetic architecture of the human cerebral cortex. Science. 2020;367:y6690. 10.1126/science.aay6690 PMC7295264 32193296

[18] Desikan RS Segonne F Fischl B. An automated labeling system for subdividing the human cerebral cortex on MRI scans into gyral based regions of interest. Neuroimage. 2006;31:968–80. 16530430 10.1016/j.neuroimage.2006.01.021

[19] Hibar DP Stein JL Renteria ME. Common genetic variants influence human subcortical brain structures. Nature. 2015;520:224–9. 25607358 10.1038/nature14101 PMC4393366

[20] Wang Y Li T Fu L Yang S Hu Y-Q. A novel method for Mendelian randomization analyses with pleiotropy and linkage disequilibrium in genetic variants from individual data. Front Genet. 2021;12:634394. 34322150 10.3389/fgene.2021.634394 PMC8312241

[21] Pierce BL Ahsan H Vanderweele TJ. Power and instrument strength requirements for mendelian randomization studies using multiple genetic variants. Int J Epidemiol. 2011;40:740–52. 20813862 10.1093/ije/dyq151 PMC3147064

[22] Verbanck M Chen CY Neale B Do R. Detection of widespread horizontal pleiotropy in causal relationships inferred from Mendelian randomization between complex traits and diseases. Nat Genet. 2018;50:693–8. 29686387 10.1038/s41588-018-0099-7 PMC6083837

[23] Burgess S Thompson SG. Interpreting findings from Mendelian randomization using the MR-Egger method. Eur J Epidemiol. 2017;32:377–89. 28527048 10.1007/s10654-017-0255-x PMC5506233

[24] Bowden J Davey SG Haycock PC. Consistent estimation in Mendelian randomization with some invalid instruments using a weighted median estimator. Genet Epidemiol. 2016;40:304–14. 27061298 10.1002/gepi.21965 PMC4849733

[25] Bowden J Davey SG Burgess S. Mendelian randomization with invalid instruments: effect estimation and bias detection through Egger regression. Int J Epidemiol. 2015;44:512–25. 26050253 10.1093/ije/dyv080 PMC4469799

[26] Liu Z Kemp TJ Gao YT. Association of circulating inflammation proteins and gallstone disease. J Gastroenterol Hepatol. 2018;33:1920–4. 29671891 10.1111/jgh.14265 PMC7576672

[27] Nikolopoulos D Manolakou T Polissidis A. Microglia activation in the presence of intact blood-brain barrier and disruption of hippocampal neurogenesis via IL-6 and IL-18 mediate early diffuse neuropsychiatric lupus. Ann Rheum Dis. 2023;82:646–57. 36898766 10.1136/ard-2022-223506 PMC10176423

[28] Li K Qu H Ma M. Correlation between brain structure atrophy and plasma amyloid-beta and phosphorylated tau in patients with Alzheimer’s disease and amnestic mild cognitive impairment explored by surface-based morphometry. Front Aging Neurosci. 2022;14:816043. 35547625 10.3389/fnagi.2022.816043 PMC9083065

[29] Velasquez C Gomez E Martino J. Mapping visuospatial and self-motion perception functions in the left parietal lobe. Neurosurg Focus. 2018;45:V8. 30269556 10.3171/2018.10.FocusVid.18286

[30] Yang H Xu H Li Q. Study of brain morphology change in Alzheimer’s disease and amnestic mild cognitive impairment compared with normal controls. Gen Psychiatr. 2019;32:e100005. 31179429 10.1136/gpsych-2018-100005 PMC6551438

[31] Susianti NA Prodjohardjono A Vidyanti AN. The impact of medial temporal and parietal atrophy on cognitive function in dementia. Sci Rep. 2024;14:5281. 38438548 10.1038/s41598-024-56023-3 PMC10912680

[32] Patra A Kaur H Chaudhary P Asghar A Singal A. Morphology and morphometry of human paracentral lobule: an anatomical study with its application in neurosurgery. Asian J Neurosurg. 2021;16:349–54. 34268163 10.4103/ajns.AJNS_505_20 PMC8244697

[33] Zhang R Zhang L Wei S. Increased amygdala-paracentral lobule/precuneus functional connectivity associated with patients with mood disorder and suicidal behavior. Front Hum Neurosci. 2020;14:585664. 33519398 10.3389/fnhum.2020.585664 PMC7843440

[34] Brynska A Wolak T Naumczyk P Srebnicki T Wolańczyk T. Morphometric evaluations based on voxel based morphometry on adolescents with autism spectrum disorders. Psychiatr Pol. 2022;56:1049–59. 37074856 10.12740/PP/OnlineFirst/132704

[35] Chindaprasirt J Sookprasert A Sawanyawisuth K Limpawattana P Tiamkao S. Brain metastases from cholangiocarcinoma: a first case series in Thailand. Asian Pac J Cancer Prev. 2012;13:1995–7. 22901160 10.7314/apjcp.2012.13.5.1995

[36] Schmidt SL Schmidt JJ Tolentino JC. Cholangiocarcinoma associated with limbic encephalitis and early cerebral abnormalities detected by 2-deoxy-2-[fluorine-18]. fluoro-D-glucose integrated with computed tomography-positron emission tomography: a case report. J Med Case Rep. 2016;10:200. 27439460 10.1186/s13256-016-0989-1 PMC4955157

[37] Zhong X Liao Y Chen X. Abnormal serum bilirubin/albumin concentrations in dementia patients with abeta deposition and the benefit of intravenous albumin infusion for Alzheimer’s disease treatment. Front Neurosci. 2020;14:859. 33013289 10.3389/fnins.2020.00859 PMC7494757

[38] Tang WK Liang H Chu WC Mok V Ungvari GS Wong KS. Association between high serum total bilirubin and post-stroke depression. Psychiatry Clin Neurosci. 2013;67:259–64. 23683157 10.1111/pcn.12051

[39] Wang X Wu W Hou BL. Studying neonatal bilirubin encephalopathy with conventional MRI, MRS, and DWI. Neuroradiology. 2008;50:885–93. 18563403 10.1007/s00234-008-0423-5

[40] Yu G Liu Z Wu X. Common and disorder-specific cortical thickness alterations in internalizing, externalizing and thought disorders during early adolescence: an adolescent brain and cognitive development study. J Psychiatry Neurosci. 2023;48:E345–56. 37673436 10.1503/jpn.220202 PMC10495167

[41] Uellendahl-Werth F Maj C Borisov O. Cross-tissue transcriptome-wide association studies identify susceptibility genes shared between schizophrenia and inflammatory bowel disease. Commun Biol. 2022;5:80. 35058554 10.1038/s42003-022-03031-6 PMC8776955

[42] Knussmann GN Anderson JS Prigge M. Test-retest reliability of FreeSurfer-derived volume, area and cortical thickness from MPRAGE and MP2RAGE brain MRI images. Neuroimage Rep. 2022;2:100086. 36032692 10.1016/j.ynirp.2022.100086 PMC9409374

[43] Yan S Qian T Maréchal B. Test-retest variability of brain morphometry analysis: an investigation of sequence and coil effects. Ann Transl Med. 2020;8:12. 32055603 10.21037/atm.2019.11.149 PMC6995743

