# Supplementary material for: A Mendelian randomization study exploring the genetic associations between biliary system disorders and brain structural changes
Source: Medicine (Baltimore). 2026 Feb 13;105(7):e47616. doi: 10.1097/MD.0000000000047616 (PMC12908830; doi:10.1097/MD.0000000000047616)
Supplement: Supplementary file 2 [file medi-105-e47616-s002.pdf]

Figure S1. Scatter plots illustrating the causality between biliary tract diseases and the cerebral cortex.

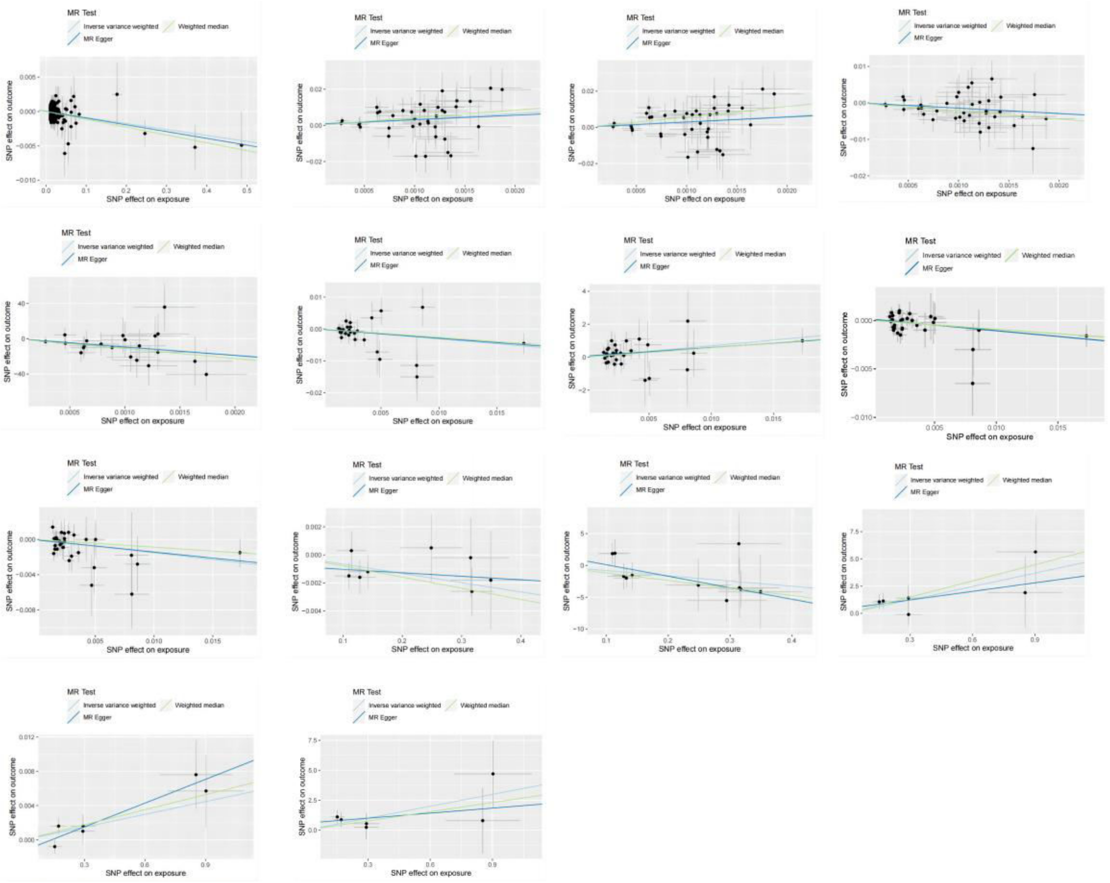

(A) Total bilirubin (TBIL) and total hippocampal (TH) volume of the pars opercularis with global. (B) Intrahepatic cholangiocarcinoma (ICC) and TH of the parahippocampal gyrus without global. (C) ICC and total hippocampal volume without global. (D) ICC and TH of the pars opercularis without global. (E) ICC and surface area (SA) of the superior parietal lobule without global. (F) Gallstone disease (GSD) and TH of the transverse temporal gyrus without global. (G) GSD and SA of the transverse temporal gyrus with global. (H) GSD and TH of the inferior parietal lobule with global. (I) GSD and TH of the middle temporal gyrus

with global. (J) Cholecystitis and TH of the paracentral lobule with global. (K) Cholecystitis and SA of the paracentral lobule without global. (L) Primary sclerosing cholangitis (PSC) and SA of the parahippocampal gyrus with global. (M) PSC and TH of the paracentral lobule with global. (N) PSC and SA of the parahippocampal gyrus without global.

Figure S2. Leave-one-out plots of biliary tract diseases-cerebral cortex causality.

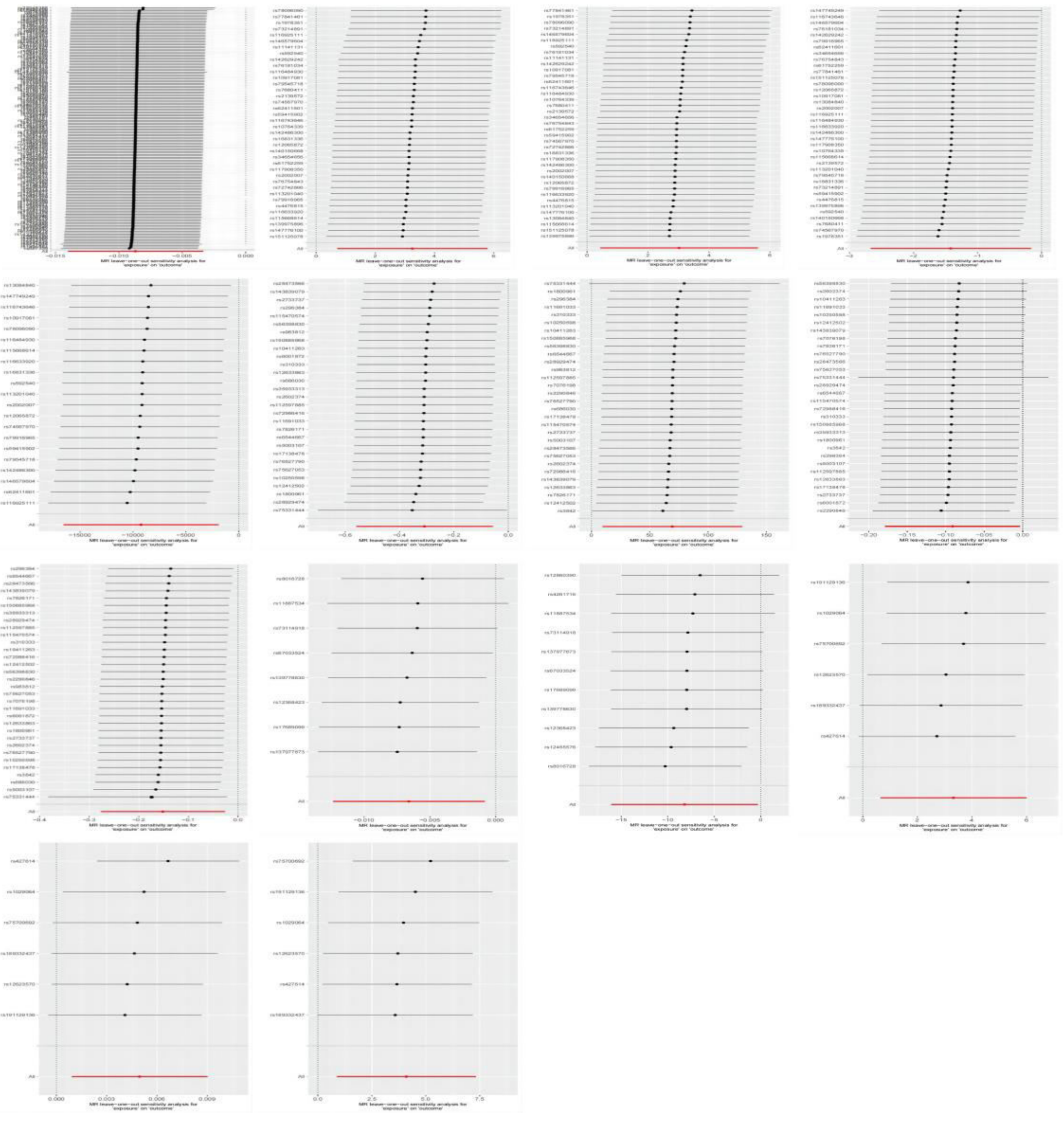

Figure S3. Funnel plots of biliary tract diseases-cerebral cortex causality.

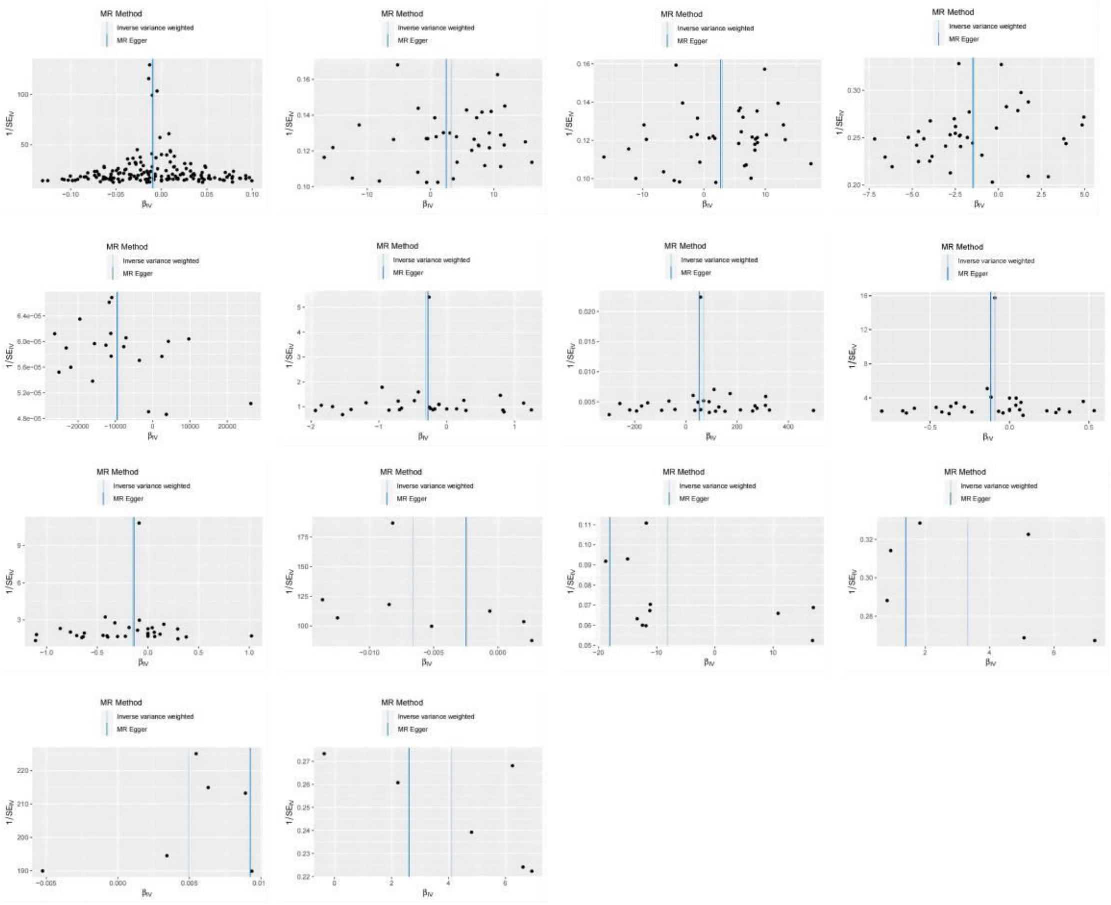

Table S1. Genetically predicted cortical structures from TBIL, ICC, PSC, GSD, and cholecystitis were evaluated using nominal Mendelian randomization.

Exposures<br>Outcomes	Method	β(95% CI)	SE	P value
TBIL				
Surface area of full cortex<br>Thickness of full cortex	IVW	-588.4351(-1458.5934 to 281.7231)<br>0.0019 (-0.0035 to 0.0075)	443.9582<br>0.0028	0.19<br>0.48
PSC				
Surface area of full cortex<br>Thickness of full cortex	IVW	221.7117(-294.9404 to 738.3638)<br>0.0018(-0.0016 to 0.0052)	263.5980<br>0.0017	0.40<br>0.32
cholecystitis				
Surface area of full cortex<br>Thickness of full cortex	IVW	-306.0843(-903.6009 to 291.4323)<br>0.0012(-0.0034 to 0.0058)	304.8554<br>0.0023	0.31<br>0.62
ICC				
Surface area of full cortex<br>Thickness of full cortex	IVW	28173.8617(-109508.4193 to 165856.1427)<br>-0.4220(-1.3060 to 0.4620)	70246.0617<br>0.4510	0.69<br>0.35
GSD				
Surface area of full cortex<br>Thickness of full cortex	IVW	-10761.8975(-27585.0787 to 6061.2836)<br>0.0267(-0.0905 to 0.144)	8583.2557<br>0.0598	0.21<br>0.65

Table S2. Nominal Mendelian randomization assessments from total bilirubin, ICC, GSD, and cholecystitis on genetic prediction of subcortical brain structures.

Exposures<br>Outcomes	Method	β (95% CI)	SE	P value
TBIL				
ICV	IVW	12080.6(-20963.32 to 45124.53)	16859.14	0.47
accumbens		-5.33(-25.31 to 14.65)	10.19	0.60
amygdala		31.69(-13.98 to 77.36)	23.30	0.17
caudate		-40.43(-127 to 46.14)	44.17	0.36
hippocampus		40.42(-36.88 to 117.72)	39.44	0.31
pallidum		1.63(-30.48 to 33.75)	16.38	0.92
putamen		-0.33(-113.23 to 112.58)	57.60	0.995
thalamus		-18.77(-130.09 to 92.55)	56.80	0.74
cholecystitis				
ICV	IVW	-4983.85(-22039.7 to 12072.10)	8702.01	0.57
accumbens		-1.31(-15.13 to 12.52)	7.05	0.85
amygdala		4.26(-27.83 to 36.35)	16.37	0.79
caudate		14.79(-30.34 to 59.91)	23.02	0.52
hippocampus		-26.80(-70.95 to 17.36)	22.53	0.23
pallidum		-0.74(-18.59 to 17.12)	9.11	0.94
putamen		-6.74(-61.74 to 48.27)	28.06	0.81
thalamus		-8.47(-94.27 to 77.34)	43.78	0.85
ICC				
ICV	IVW	218737.36(-7914112.48 to 8351587.20)	4149413.19	0.96
accumbens		649.95(-7038.46 to 8338.36)	3922.66	0.87
amygdala		1920.30(-8728.29 to 12568.88)	5432.95	0.72
caudate		-7842.09(-29362.11 to 13677.93)	10979.60	0.48
hippocampus		9727.95(-11320.55 to 30776.46)	10739.03	0.37
pallidum		6949.10(-1451.23 to 15349.43)	4285.88	0.10
putamen		8785.59(-2479.67 to 18945.39)	8977.56	0.75
thalamus		4247.86(-23385.57 to 31881.30)	14098.69	0.76
GSD				
ICV	IVW	322319.39(-683728.1 to 1328366.88)	513289.54	0.53
accumbens		-226.67(-804.71 to 351.38)	294.92	0.44
amygdala		947.54(-289.61 to 2184.7)	631.20	0.13
caudate		-26.03(-2592.26 to 2540.2)	1309.30	0.98
hippocampus		360.15(-2702.29 to 3422.59)	1562.47	0.82
pallidum		-611.34(-1682.91 to 460.23)	546.72	0.26
putamen		-470.44(-4110.99 to 3170.11)	1857.42	0.80
thalamus		-2327.02(-5622.1 to 968.06)	1681.16	0.17
